# Supplementary material for: A Mechanistic Study of Asymmetric Transfer Hydrogenation of Imines on a Chiral Phosphoric Acid Derived Indium Metal-Organic Framework
Source: Molecules. 2022 Nov 26;27(23):8244. doi: 10.3390/molecules27238244 (PMC9738091; doi:10.3390/molecules27238244)
Supplement: Supplementary file 1 [file molecules-27-08244-s001.zip › molecules-2043141-supplementary.pdf]

Electronic supplementary information

A Mechanistic Study of Asymmetric Transfer Hydrogenation of Imines on a Chiral Phosphoric Acid Derived Indium Metal-Organic Framework

Xu Li*[a], Ting Fan[c], Qingji Wang *[b] and Tongfei Shi *[a]

[a]*School of Light Chemical Engineering, Guangdong University of Technology, Guangzhou, Guangdong 510006, China.*
[b]*College of Information and Communication Engineering, Hainan University, Haikou 570228, China.*
[c]*School of Chemistry and Chemical Engineering, South China University of Technology, Guangzhou 510641, China.*

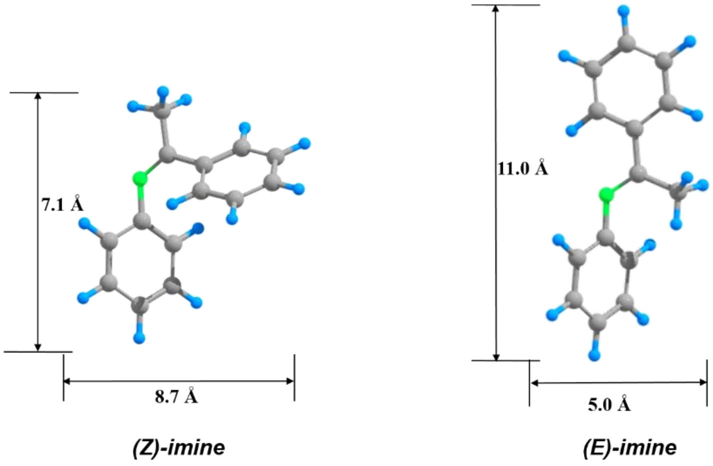

Figure. S1. Molecular dimensions of (***Z***)- and (***E***)-imine. C: grey, N: green, H: blue.

Table S1. Imaginary frequencies (i cm^{-1}) for transition states on the In-MOF in toluene.

−H		−OCH_3		−F		−NO_2	
**TS-*R***	**TS-*S***	**TS-*R***	**TS-*S***	**TS-*R***	**TS-*S***	**TS-*R***	**TS-*S***
-903.91	-992.45	-950.03	-972.01	-841.95	-946.02	-1054.32	-611.32

Table S2. Electronic energies (E^{ele}) and thermal corrections at 60 °C (G^{therm}) of cluster, thiazoline, imine, co-adsorption complex (co-ad), transition state, and product on the In-MOF in toluene. The unit is Hartree.

	G^{therm}	E^{ele}			
	B3LYP	B3LYP-D3	M06-2X	M06-L	ωB97XD
cluster	0.172806	-1176.747919	-1173.118950	-1173.372676	-1173.203436
thiazoline	0.118324	-801.474893	-801.103497	-801.236925	-801.162826
(***Z***)-imine	0.184796	-596.288696	-595.833473	-596.016802	-595.896952
(***E***)-imine	0.184777	-596.282544	-595.836788	-596.021149	-595.899453
co-ad	0.531890	-2574.728930	-2570.168325	-2570.738474	-2570.384150
**TS-*R***	0.533730	-2574.719330	-2570.155003	-2570.737330	-2570.370950
product	0.535400	-2574.762000	-2570.203596	-2570.777020	-2570.414620
co-ad	0.536324	-2574.73097	-2570.164333	-2570.739584	-2570.381182
**TS-*S***	0.535495	-2574.71564	-2570.147913	-2570.731563	-2570.363593
product	0.533537	-2574.75582	-2570.201611	-2570.776986	-2570.414252

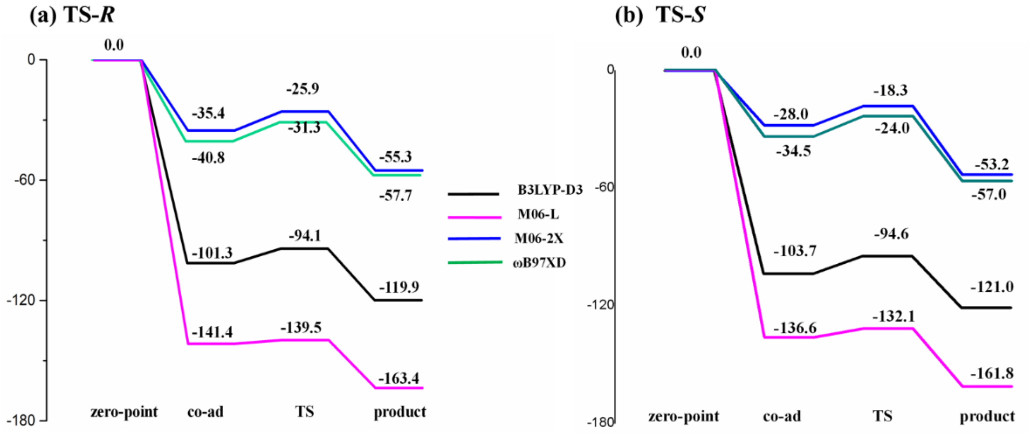

Figure S2. Relative Gibbs energies (kcal/mol) *via* (a) **TS-*R*** (b) **TS-*S*** on the In-MOF in toluene calculated by different functionals.

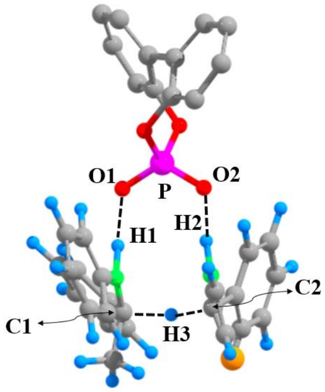

Figure S3. Selected distances between transition state and CPA. O1 and O2: oxygen atoms in CPA; H1 and C1: hydrogen and carbon atoms in imine; H2, H3 and C2: hydrogen and carbon atoms in thiazoline. The transition state shown is **TS-*R*.** Color code: P, pink; O, red; N, green; C, grey; S, orange and H, blue.

Table S3. Selected distances (Å) for transition states on the In-MOF in toluene.

Distance	−H		$-OCH_3$		−F		$-NO_2$	
	**TS-*R***	**TS-*S***	**TS-*R***	**TS-*S***	**TS-*R***	**TS-*S***	**TS-*R***	**TS-*S***
O1-H1	1.65	1.93	1.65	1.94	1.64	1.93	1.65	1.97
O2-H2	1.61	1.63	1.60	1.60	1.61	1.61	1.56	1.55
C1-H3	1.50	1.45	1.48	1.44	1.51	1.45	1.40	1.29
C2-H3	1.31	1.33	1.32	1.33	1.30	1.32	1.37	1.38

Table S4. Electronic energies (E^{ele}) and thermal corrections at 60 °C (G^{therm}) of imine (R = $-NO_2$, −F and $-OCH_3$), co-adsorption complex (co-ad), transition state, and product on the In-MOF in toluene. The unit is Hartree.

	$-NO_2$		−F		$-OCH_3$	
	G^{therm}	E^{ele}	G^{therm}	E^{ele}	G^{therm}	E^{ele}
(***Z***)-imine	0.182250	-800.346045	0.175154	-695.111111	0.213231	-710.390566
(***E***)-imine	0.182091	-800.348492	0.175315	-695.113257	0.213724	-710.392755
co-ad	0.528810	-2774.833010	0.522470	-2671.477580	0.563710	-2684.885370
**TS-*R***	0.532370	-2774.825890	0.524260	-2671.463870	0.562920	-2684.869340
product	0.533960	-2774.874030	0.526610	-2671.506570	0.563340	-2684.909850
co-ad	0.534390	-2774.824030	0.528270	-2671.474200	0.568040	-2684.879580
**TS-*S***	0.535590	-2774.809760	0.526030	-2671.451800	0.565190	-2684.855600
product	0.530970	-2774.865690	0.523390	-2671.506360	0.562200	-2684.907150

Table S5. Electronic energies (E^{ele}) and thermal corrections at 60 °C (G^{therm}) of co-adsorption complex (co-ad), transition state, and product on the In-MOF in different solvents. The unit is Hartree.

Solvent	dichloromethane		acetonitrile		dimethylsulfoxide	
	G^{therm}	E^{ele}	G^{therm}	E^{ele}	G^{therm}	E^{ele}
co-ad	0.563710	-2684.894850	0.563710	-2684.899490	0.563710	-2684.899960
**TS-*R***	0.562920	-2684.877350	0.562920	-2684.881340	0.562920	-2684.881750
product	0.563340	-2684.917180	0.563340	-2684.920870	0.563340	-2684.921230
co-ad	0.568040	-2684.889890	0.568040	-2684.894900	0.568040	-2684.895400
**TS-*S***	0.565190	-2684.864530	0.565190	-2684.869370	0.565190	-2684.869880
product	0.562200	-2684.914970	0.562200	-2684.919230	0.562200	-2684.919680

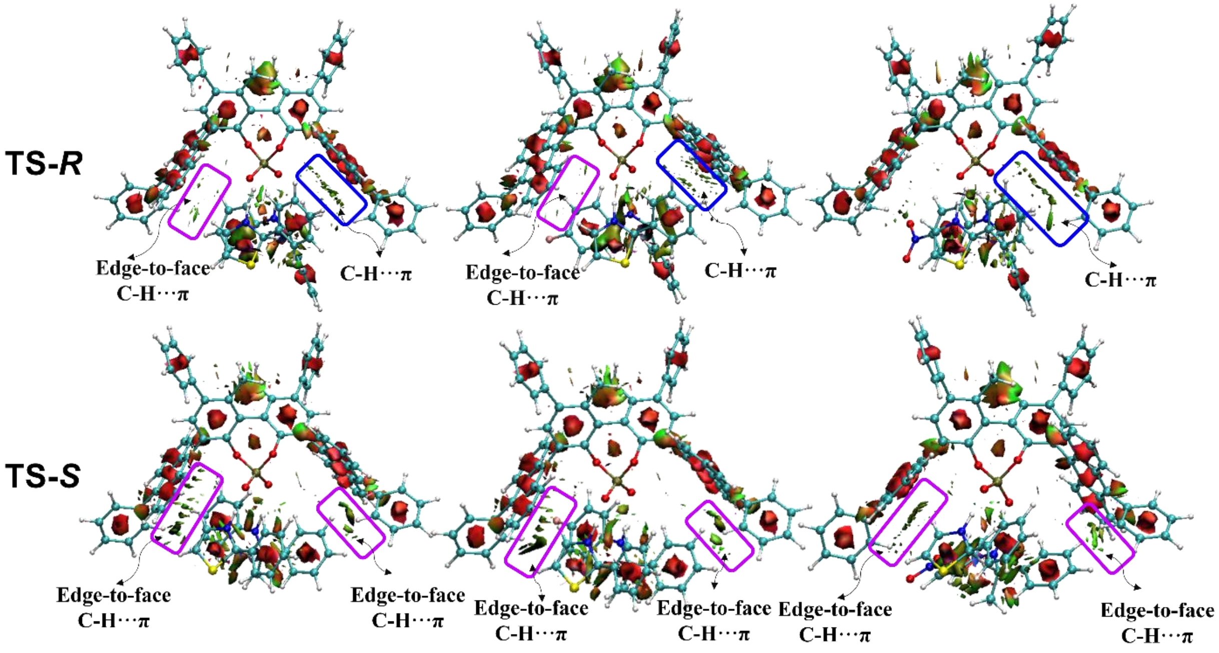

Figure S4 NCI analysis of TSs with −H, $-NO_2$, −F substituted imines (blue, strong attraction; green, weak interaction; red, strong repulsion). The pink and blue cycles represent the steric effects due to edge-to-face C-H···π and C-H···π interactions, respectively.
